# Typological thinking: Then and now

**DOI:** 10.1002/jez.b.22796

**Published:** 2018-03-26

**Authors:** Joeri Witteveen

**Affiliations:** ^1^ Descartes Centre for the History and Philosophy of the Sciences and the Humanities Utrecht University Utrecht The Netherlands; ^2^ Department of Science Education Section for History and Philosophy of Science University of Copenhagen Copenhagen Denmark

**Keywords:** Bauplan, body plans, Ernst Mayr, Essentialism Story, George Gaylord Simpson, morphological type, phyla, typological thinking, typology

## Abstract

A popular narrative about the history of modern biology has it that Ernst Mayr introduced the distinction between “typological thinking” and “population thinking” to mark a contrast between a metaphysically problematic and a promising foundation for (evolutionary) biology, respectively. This narrative sometimes continues with the observation that, since the late‐20th century, typological concepts have been making a comeback in biology, primarily in the context of evolutionary developmental biology. It is hard to square this narrative with the historical and philosophical literature on the typology/population distinction from the last decade or so. The conclusion that emerges from this literature is that the very distinction between typological thinking and population thinking is a piece of mere rhetoric that was concocted and rehearsed for purely strategic, programmatic reasons. If this is right, it becomes hard to make sense of recent criticisms (and sometimes: espousals) of the purportedly typological underpinnings of certain contemporary research programs. In this article, I offer a way out of this apparent conflict. I show that we can make historical and philosophical sense of the continued accusations of typological thinking by looking beyond Mayr, to his contemporary and colleague George Gaylord Simpson. I show that before Mayr discussed the typology/population distinction as an issue in scientific metaphysics, Simpson introduced it to mark several contrasts in methodology and scientific practice. I argue that Simpson's insightful discussion offers useful resources for classifying and assessing contemporary attributions of typological thinking.

## 1 THE MAKING OF “TYPOLOGICAL THINKING”

A popular narrative about an episode in the history of evolutionary biology from the mid‐20th century to today runs as follows: In the heyday of the modern evolutionary synthesis, Ernst Mayr (1904–2005) coined the term “typological thinking” for a collection of misguided metaphysical ideas and anti‐evolutionary commitments that were shared by many morphologists, anatomists, and paleontologists. Their reliance on concepts of “body plans” and “morphological types” suggested an ontology of idealistic, otherworldly forms—an ontology for which there was no place in modern, Darwinian biology (Mayr, 1959). The alternative to this typological mode of thinking was the “population thinking” of the modern synthesis: an ontology and outlook that eschewed postulating the existence of (supra)specific types and that constrained the study of evolution to variants of genes, alleles, and genotypes in populations and species. This narrative about how typological thinking was supplanted by population thinking is sometimes continued with the observation that, since the home stretch of the 20th century, there has been a gradual resurgence of talk about body plans, morphological types, and phylotypic stages, primarily in the context of evolutionary developmental biology (e.g., Amundson, 2005; Wagner, 2014). According to some, this resurgence of typological language has been accompanied by an unwelcome return of what remains at heart a static, antievolutionary, and hence “unbiological” approach to biology. But others argue that it is high time for a reevaluation of typological thinking, which may provide helpful and perhaps even indispensable resources for the project of explaining the origin of form. According to them, the modern synthesis has unduly sidelined and marginalized fruitful scientific styles and approaches, by branding all theorizing about “types” as mystical and unscientific.

Popular as this narrative may be, it has become problematic in the light of the historical and philosophical literature on the typology/population distinction from the last decade or so. The conclusion that emerges from this literature is that the very distinction between typological thinking and population thinking is a piece of mere rhetoric that was devised for strategic and programmatic purposes, primarily by Mayr (Winsor, 2003; Levit et al., 2006). For example, historian of biology Polly Winsor has argued that Mayr's attack on typological thinking from the late 1950s onward was “an enormously effective bit of polemic” meant to sideline critics of the modern synthesis “rather than accurately describe the avowed position of anyone” (Winsor, 2006, p. 159). The same holds, according to Winsor, for Mayr's later accusations of “essentialism,” a notion he tended to equate with “typology” and “typological thinking.” Mayr thus successfully created and disseminated a mythical “Essentialism Story” that portrayed the history of biology as an ongoing clash between typologists and population thinkers, culminating with the victory of the latter camp in the modern synthesis (Winsor, 2003; Wilkins, 2009).

But if the notion of typological thinking that Mayr and others were up against was indeed a fabrication, what should we make of recent criticisms (and sometimes: espousals) of the purportedly typological underpinnings of certain contemporary research programs? According to the revisionist historiography these revived claims of typology must be idle and meaningless. For example, in a review of an edited volume on recent developments in evo‐devo, Rieppel (2008) observed that although some practitioners of evo‐devo “still feel haunted by the neo‐Darwinian charge of typological thinking … it is time to recognize the charges of ‘typology’ and ‘typological thinking’ for what they are – creatures of ‘the Essentialism Story’” (p. 506).

In this essay, I will argue that it is nevertheless worth salvaging a notion of typological thinking from the scrapheaps of the Essentialism Story. I will show that although Winsor and others were right about the opacity of Mayr's accusations of typological thinking, they largely overlooked that these had descended (with considerable modification) from an earlier, far more substantive critique of typological thinking due to George Gaylord Simpson (1902–1984). In contrast with Mayr, Simpson's discussions of typology were not focused on metaphysical concerns, but instead issued from clearly articulated methodological and conceptual matters in the taxonomic practice of his day. Recovering Simpson's thoughts on typological thinking is important because it can provide a bridge to contemporary discussions in which some of his concerns continue to be echoed.

I will use Simpson's discussions to distinguish between three different “grades” of typological thinking that he took issue with, before showing that attributions of each of these grades can also be discerned in contemporary debates at the interface of paleontological systematics and evo‐devo.^1^ I will argue that although these attributions of typological thinking are meaningful as such, they often miss their mark in the contemporary context. Many of the errors of typological thinking that Simpson identified back in his day no longer apply today.

## 2 TYPOLOGY THEN: SIMPSON ON SYSTEMATICS

The origins of what would develop into the distinction between typological and population thinking can be traced to 1937. In that year Simpson wrote two articles on methods and principles of systematics (Simpson, 1937a,b), and Theodosius Dobzhansky published his soon‐to‐be‐famous monograph *Genetics and the Origin of Species* (Dobzhansky, 1937). In these publications, Simpson and Dobzhansky independently began to criticize a notion of types and made a case for what they characterized as a population‐based approach. However, since these two men were concerned with different notions of types that featured in different contexts, they ended up articulating different kinds of type/population distinctions. Starting in the 1950s, Mayr began to interweave these distinct type/population contrasts into a single, overarching dichotomy. Elsewhere, I have argued that what Mayr took to be a successful attempt at integration resulted in the confounding of importantly different issues (Witteveen, 2015, 2016a). In his insistence that the typology/population distinction was all about the metaphysics of biology, Mayr obfuscated the various practical, methodological and theoretical concerns that had been at the center of Simpson's and Dobzhansky's original discussions of type concepts in biology (Mayr, 1959, 1982). I will not here recapitulate this complex account of the transformation and popularization of the typology/population distinction by Mayr. Instead, I will discuss a particular *strand* of this history that originated with Simpson. I will argue that Simpson's writings on types and typology can be used to shed light on the meaning and force of several contemporary attributions of typological thinking.

The two 1937 articles that inaugurated Simpson's concern with things typological were “Patterns of Phyletic Evolution” (Simpson, 1937a) and “Supra‐specific variation in nature and in classification from the viewpoint of paleontology” (Simpson, 1937b). Although Simpson did not use the *term* “type” or any of its derivatives in either of these articles, it is clear that they started orienting him toward a critique of methods and principles that he would later associate with a notion of types. When Simpson reflected on the first of these two articles toward the end of his scientific career, he reminisced that it had marked the “abandonment of typological thinking of my college teachers and started aiming me toward statistical biometry and the deeper investigation of evolutionary theory and taxonomic stance” (Simpson, 1978, p. 112). What was this “typological thinking” that Simpson had abandoned?

A closer look at the “Patterns” article reveals a discussion of an approach to classification based on methods, rules, and standards that Simpson considered to be outdated scientifically. As an example of this approach Simpson mentioned a classificatory rule he had been taught in graduate school: “If one specimen is as much as 15 percent larger than another in any linear dimension, it is safe enough to assume that they belong to different species” (Simpson, 1937a, p. 307). Simpson noted that, in practice, every taxonomist recognized that this was merely a rule of thumb: in some cases it could be perfectly valid to assign two specimens to the same species if they differed by more than 15%, in other cases a sound taxonomic judgment assigned specimens to different species even if they differed by much less than 15%. However, Simpson argued that even this looser interpretation of the “15 percent rule” as a first‐pass heuristic was problematic, because of the more general practice of grouping organisms into species that it was associated with. Taxonomists tended to attribute new specimens to one or the other species by comparing them in a one‐by‐one fashion with representative specimens of several known species and by judging which of these representatives it resembled most. Simpson objected that this practice of classification meant that valuable information about the range of variation within species was ignored. This may have once been necessary, but it was no longer excusable now that sophisticated methods of inferential statistics were available. Using these statistical techniques, taxonomists could now compare entire samples of specimens, instead of relying on individual specimens that stood in for their groups.

Simpson gave examples of how inferential statistics could be applied to taxonomy in two other publications from the same year (Simpson, 1937c,d), as he started coauthoring a monograph on statistical methods in zoology with his partner Anne Roe (Simpson & Roe, 1939). In this monograph, Simpson and Roe for the first time addressed the relation between the method of one‐by‐one comparison and a notion of “types.” Having observed that many taxonomists took so‐called “type specimens” to be key to the practice of one‐by‐one comparison, they pointed out that this use of type specimens as standards of comparison amounted to a double error. It compounded the use of a statistically deficient method of classification with a flawed understanding of the one and only role that type specimens should serve in taxonomy.

The modern notion of a type specimen was not that of a typical exemplar that could function as a standard of comparison, but instead referred to the unique name‐bearing specimen of a species. In the act of describing a new species, it had become common practice to select one specimen as the permanent anchor for the new species name. This use of fixed specimens as anchors for species names enabled any two taxonomists to agree about which hypothesized species circumscription should be associated with which species name, even if they disagreed about whether the circumscription correctly identified the boundaries of the named species.^1^ However, this use of type specimens implied that they could not be used as representative exemplars of their species’ typical traits. Even if a specimen had seemed representative of a species at the time it was selected as that species’ name‐bearer, it could easily stop being representative after a revision of the species’ hypothesized circumscription. For purposes of classification, a type specimen was on a par with any other specimen. For purposes of naming, knowing which type specimen was part of which hypothesized circumscription was essential.

Simpson neatly summarized this lesson about the proper use of name‐bearing types into an explicit contrast between types and populations. He pointed out that modern taxonomic practice should be “based on inferences about populations from samples, a view that is rapidly gaining ground and to which I strongly adhere, is decisively incompatible with this use of types [as standards of comparison]” (Simpson, 1940; reprinted in Simpson, 1981). It is worth noting the methodological and eminently practice‐oriented nature of this type/population distinction—a far cry from the metaphysical dichotomy that Mayr would popularize years later.

Simpson's concerns with types did not end here. In the second important article from 1937, he laid the groundwork for a closely related discussion of types that will be of primary interest to us (Simpson, 1937b). The aim of this article was to assess and elaborate on a series of principles and methods for the classification of higher taxa that had recently been outlined by the entomologist Alfred Kinsey (1936). One principle from Kinsey that Simpson highlighted was the idea that higher taxa should not be delimited by searching for unique sets of characters common to a group of organisms. A higher taxon was to be defined “not by a fixed set of characters at any given point in time but by the transmutation of its characters throughout its history, a fluid character embracing the group as a whole and always distinctive from any similar related group” (ibid., p. 255).

Taken at face value, these observations about supra‐specific classification may seem unrelated to Simpson's writings on the methodology of classification at the species level, or to the notion of types he discussed in that context. But in subsequent writings Simpson made clear that these two discussions were closely related after all. He noted that his main criticism of the “characters‐in‐common approach” to higher classification was essentially the same as his objection to the use of classificatory types at the species level. In both contexts, he was taking issue with the appointment of standards of comparison that supposedly picked out the typical or representative features of a group, and that could be used to pass judgment on what else belonged in the group. Simpson argued that by following this practice of classification, taxonomists were effectively confusing *diagnosis*, the task of giving “a statement of the difference between adjacent groups,” with *definition*, the task of providing “a description of the characters and limits of variation of a single group” (Simpson, 1943 p. 152). Simpson emphasized that the latter task was evidently epistemically prior to the former—a good estimate of a group's boundaries was needed before (fallible) diagnostic characters could be selected to tell groups apart. Yet many taxonomists were putting the cart before the horse. They helped themselves to purportedly diagnostic characters and then used these to hypothesize about taxon limits.

In formulating an alternative approach to determining the limits of higher taxa, Simpson gave further substance to the connection with his type/population distinction at the species level. He repeatedly stressed that the use of classificatory rules‐of‐thumb at any taxonomic level impeded a proper consideration of the spectrum of variation and could therefore result in flawed classifications. He sometimes characterized the inflexible classificatory standards as “archetypal” or “typological” models, incapable of representing the variational nature of taxonomic groups (Simpson, 1941, 1945, 1951). Modern taxonomy, he argued, should instead be “statistical in the broad sense,” by which he meant that it should be rooted in “(a) estimating the characteristics of populations from samples; and (b) describing groups, as such, rather than individuals taken singly” (Simpson, 1943, p. 151). This two‐pronged characterization of statistics is noteworthy. It illustrates that Simpson was not merely advocating the use of mathematical methods and the use of larger quantities of numerical data—he emphasized that “good taxonomic work can be and is being done without them” (ibid., p. 151)—but that, instead, he wanted to drive home that all inferences to the limits of taxonomic groups should be rooted in a conception of those groups as intrinsically variational units.

## 3 THREE GRADES OF TYPOLOGICAL THINKING

We have seen that the picture of typological thinking that emerges from Simpson's two 1937 articles and their follow‐ups is that of an approach to biological systematics with problematic methodological and conceptual dimensions. In advocating population‐based over type‐based approaches to classification, Simpson combined a plea for the use of novel statistical methods with the subtler conceptual point that conventional approaches had led many a taxonomist to confuse the job of defining (i.e., determining the limits of) taxa with that of diagnosing them (i.e. telling taxa apart).

But Simpson did not stop here. In subsequent writings, he pointed out that what we might call this *conceptual‐methodological* mode of typological thinking could serve as a platform for sliding into other, more pernicious forms of typological thinking. We can distinguish two other “grades” of typological thinking that Simpson discussed: *logical* typological thinking and *theoretical‐empirical* typological thinking.

The lapse from conceptual‐methodological into logical typological thinking is demonstrated in Simpson's discussion of taxonomic ranks. If one had already failed to distinguish properly between diagnosis and definition (i.e., by taking sets of characters‐in‐common to be defining of taxa), one would be but a small step away from thinking that different *kinds* of characters could be defining of taxa at different *ranks*. However, Simpson pointed out, this way of thinking about taxonomic ranks was obviously flawed. A character that is diagnostic of, say, a subspecies at one point in time can easily become diagnostic of a species if the original group is turned into a species by isolation, and it can become diagnostic of a new genus after further divergence. “It is inevitable that diagnostic characters should thus appear as individual variations and tend gradually to become subspecific, specific, then generic characters, and that no particular kind of character should be characteristic of a particular taxonomic level” (ibid., p. 157).

The error of viewing certain characters as strictly rank‐defining was part and parcel of an erroneous view about the nature of higher taxa that we might characterize as *logical* typological thinking: the idea that the Linnaean taxonomic hierarchy as such evolves, with taxa of higher rank “splitting” into taxa of lower rank in the course of time. Simpson pointed out that this view about the “top‐down” evolution of taxa was evidently flawed. By taxonomic necessity, any organism (dead or alive) must belong to some taxon at *all* main ranks. In the Linnaean system of classification, no organism can belong to a family without also belonging to a genus and species within that family. It was wrong to think of the Linnaean hierarchy itself as a product of evolution (Simpson, 1943). And yet, it appeared to Simpson that contemporaries like Richard Goldschmidt, Otto Schindewolf, and John Willis were making exactly this error. The adoption of logical typological thinking was perhaps best illustrated by Goldschmidt, who wrote:

A phylum consists of a number of classes all of which are basically recognizable as belonging to the phylum but, in addition, are different from each other. The same principle is repeated at each taxonomic level. All the genera of a family have in common the traits which characterize the family; e.g., all genera of penguins are penguins. But among themselves they differ from genus to genus. So it goes on down to the level of the species. Can this mean anything but that the type of the phylum was evolved first and later separated into the types of the classes, then into orders, and so on down the line? This natural, naive interpretation of the existing hierarchy of forms actually agrees with the historical facts furnished by paleontology … Thus logic as well as historical fact tells us that the big categories existed first, and that in time they split in the form of the genealogical tree into lower and still lower categories. (Goldschmidt, 1952, pp. 91–92)

Willis similarly claimed that “evolution goes on in what may be called the downward direction from family to variety” (Willis, 1940, p. 191). It was in passages like these that Simpson saw evidence for how the relatively innocuous characters‐in‐common approach to delimiting taxa could lead to seriously mistaken inferences about the logic of Linnaean group‐within‐group classification (Simpson, 1953).

Committing the error of logical typological thinking in its turn made it easy to fall into the further trap of what we might call *theoretical‐empirical* typological thinking. The quotation from Goldschmidt already hints at this third grade of typological thinking: the error of thinking that the evolutionary process must be saltational in nature. Simpson indeed suggested that many saltationist ideas about evolution had been induced by, and were ultimately grounded in, a flawed conception of the methods and principles of classification. Those who thought of higher taxa as sharing certain rank‐defining characters were naturally attracted by the idea that new taxa of descending rank must originate in a stepwise fashion. A qualitatively new rank‐defining character would need to emerge for such a step to be taken. Again, Simpson pointed out that Goldschmidt, Schindewolf, and Willis claimed exactly this in their writings on the origins of higher taxa. And again, Goldschmidt was the exemplar: “the conclusion is reached, on the basis of the facts of development, comparative morphology, paleontology, and the features of major adaptations, that the higher categories are built up not by slow accumulation of micromutations selected for different environments but by large initial macromutations which at once, by affecting early embryological processes, produce the major basic features of the new group, [which] afterwards may be improved, perfected, and diversified” (Goldschmidt, 1952, p. 93).

## 4 TYPOLOGY NOW: BODY PLANS AND THE ORIGIN OF PHYLA

With the three grades of Simpson's critique of typological thinking on the table, I will now consider whether and in what sense these attributions of typological thinking recur in contemporary debates, and weigh in on whether they are justified in the contemporary context. I do this with respect to a particular ongoing debate at the interface of paleontological taxonomy and evo‐devo: the debate over the classification and evolution of phyla. I will argue that although the accusations of typological thinking that we encounter in this context are often remarkably similar to those that Simpson made, it is far from clear that these accusations hit their mark with regard to current scientific practice.

Let us start by looking at recent variants of Simpson's conceptual‐methodological criticism, concerning the use of characters‐in‐common as a means to delimit higher taxa. Several contemporary authors have voiced similar criticisms when commenting on the relation between the notion of body plan (or Bauplan) and the assignment of phylum‐level Linnaean ranks. For example, Scholtz (2004) has argued that the notion of a body plan reveals it typological heritage as “an abstraction of commonality of characters” that is defining of a phylum‐level lineage (also see Fitch & Sudhaus, 2002). Jenner (2006) has similarly claimed that talk of body plans and phyla in the context of evo‐devo “is characterized by a remarkable degree of typological thinking” because it “treats taxa as classes.” These claims resonate with Simpson's point about the confusions between definition and diagnosis: one should not mistake diagnostically useful morphological patterns for being defining features of higher taxa.

However, it is hard to find clear examples of this error in actual contemporary treatments of the relation between body plans and phyla. For a start, phyla are first and foremost defined as cladistically inferred higher‐level communities of descent, with body plan criteria serving as secondary means to pick out relevantly similar groups. Freeman (2014) highlights this dual nature by offering a “two part definition” of a phylum as “(a) a higher order community of descent, and (b) the extant and fossil species that share the same body plan” (p. 221). Similarly, Arthur (2000) remarks that “it is true, of course, that the concept of a body plan is a ‘grade’ or ‘type’ issue; but it is that as well as, not instead of, a clade issue” (p. 30). In addition, it is worth noting that the contemporary notion of a phylum‐level body plan tends not be conceived of as a set of characters shared by all members of a group. For example, in his important monograph *On the Origin of Phyla* (2004), James Valentine argues that a body plan should be conceived of polythetically, that is, as “an assemblage of morphological features shared among many members of a phylum‐level group. Not all of the features that characterize a given architecture need be present in every member of the phylum, however, and many of the features may be found in other phyla; it is the assemblage of numbers of these features that is unique” (p. 33). A similar conception of body plans emerges from other recent discussions (e.g. Arthur, 2000; Collins & Valentine, 2001; Wray & Strathmann, 2002; Angelini & Kaufman, 2005).

Conceiving of body plans as assemblages does involve an element of subjectivity in their identification and delimitation, but as several authors have pointed out, this element of subjectivity should not be a ground for consigning the notion of a body plan to the waste bin of typological thinking (Valentine, 2004; Kemp, 2015). First, the subjective element inherent to Linnaean classification was of course already present in Simpson's own evolutionary taxonomy (Simpson, 1961; Nelson, 2016). Moreover, there are reasons for thinking that the application of the notion of a body plan to the phylum‐level keeps the degree of subjectivity or arbitrariness in check. The phylum‐level rank within the Linnaean hierarchy is often characterized as the highest level at which we encounter morphological gaps between groups that are sufficiently distinct to be recognizable by distinct sets of salient diagnostic characters, without being able to tell how those groups are related to each other. Phyla thus stand apart from other ranks in expressing “an admission of ignorance” regarding further higher‐level relationships between phylum‐level taxa (Telford, Budd, & Philippe, 2015, p. R877). Or, as Valentine (2004) has put it, the phylum‐level is the level at which we find the clearest separation of distinct “morphological themes” that render the phylum‐level one at which we can discern “quasi‐natural” groupings (pp. 31–32).

Recent findings in evo‐devo reinforce this view of phyla as quasi‐natural groupings. Michal Levin and colleagues have shown that there is high conservation of gene expression at the mid‐developmental stage among species that are typically classified within the same phylum, versus high divergence between species of different phyla. This suggests that the “phylotypic” stage indeed carves at the joints between morphologically delimited phyla (Levin et al, 2016; cf. Hejnol & Dunn, 2016). Another, more prominent approach to providing a quasi‐naturalistic interpretation of phyla can be found in the work of Eric Davidson and Douglas Erwin, who have suggested that the stable persistence of different kernel circuits in gene regulatory networks correlates with phylum‐level classification (Davidson & Erwin, 2006). Notice that these are not typological proposals of the sort that Scholtz (2004) or Fitch and Sudhaus (2002) object to: they do not hark back to a developmental *definition* of body plans as the basis for identifying phyla. Instead, they suggest an independent developmental underpinning of ranked groups that can be (loosely) “grafted” on an evolutionary tree inferred from extensive molecular data.

Moving on to the second, logical dimension of typological thinking, we also find contemporary instances of attributions of this grade of typological thinking. As I noted in the previous section, Simpson pointed to this error when describing how the misleading notion of rank‐specific characters could induce the mistaken idea that higher taxa evolved before lower ones. In the contemporary literature, we encounter discussions of this grade of typological thinking with a surprising twist. Although contemporary commentators agree that the idea of rank‐specific characters is mistaken, some have argued that a rejection of rank‐specificity should lead us to *accept* the idea that higher taxa evolved before lower ones. What is more, it has been argued that a rejection of the “top‐down” view of evolution itself amounts to a typological thinking!

To come to grips with this seemingly inverted attribution logical typological thinking, let us zoom in on the debate in which it surfaced—the debate over the question “Why are all phyla old?”. Raff (1996) and Valentine (1995) raised this question in their search for an explanation of why the majority of animal phyla have their origin in the Late Cambrian. Fitch and Sudhaus (2002) and West‐Eberhard (2003) responded that the question is ill‐posed. In line with Simpson on logical typological thinking, Fitch and Sudhaus argued that the “Why are all phyla old?”‐question proceeds from the false presupposition that phyla have rank‐defining characters, which, once they have emerged, constitute the origin of a new phylum. According to them, this is at odds with how we should think of Linnaean ranks in a phylogenetic context, because “the different hierarchical levels of the taxonomic system (Phylum, Class, Order, etc.) are applied *arbitrarily*. These taxonomic levels reflect relative divergence points in time … not particular differences in Bauplan. That is, the groups‐within‐groups hierarchy of taxonomy simply derives from common ancestry at more and more ancient times. Phylum divisions represent divergences that occurred earlier than Class or Order divisions within the Phylum, *regardless of the grade of difference in Bauplan*” (Fitch and Sudhaus, 2002, p. 244; italics in original). Thus, even if an indisputably novel architecture had evolved recently, it would not be ranked as a new phylum‐level body plan because of its recency. Fitch and Sudhaus conclude that “‘all phyla are old’ simply because of the hierarchical restrictions of taxonomy, not because fundamental key changes to body plans have not arisen more recently” (ibid., p. 244). West‐Eberhard arrived at a similar conclusion: “By force of phylogenetic taxonomy, *systema naturae*, all phyla are old” (West‐Eberhard, 2003, p. 615).

At first glance, these claims are suggestive of a top‐down view of classification: taxa of higher rank evolved before taxa of lower rank originated. To understand how contemporary authors could have reached this opposite conclusion compared to Simpson, we need to realize that they approach classification from a present‐oriented cladistic point of view. This view is in principle compatible with the view that at any earlier time horizon any given organism can be positioned in a taxon at all main levels of the Linnaean hierarchy. However, if we recast the position of Fitch and Sudhaus in these terms, the question “Why are all phyla old?” resurfaces as a live empirical issue. If the number of thirty‐five or so animal phyla that are recognized today has remained stable since the Late Cambrian, this raises the question why such a large share of extant phyla arose during a short period and why so few have seen the light of day since. We know from quantitative studies of disparity that the range of surviving phyla is unlikely to be explained by the extinction of intermediate forms (Foote, 1997; Erwin, 2007) and that the sudden appearance of phyla is possibly related to the origin of distinctive regional patterning mechanisms (Davidson & Erwin, 2006). All this is compatible with the view that phylogenetic depth should play a restricting role in whether or not we assign phylum‐level rank. One can legitimately ask why all phyla are old while acknowledging that geologically very recent changes in developmental architectures would never be construed as inaugurating a new phylum‐level lineage. Thus, we can conclude that the “Why are all phyla old?” question should not be denounced for being typological, since it does not rest on a misconception about the relation between evolution and the Linnaean hierarchy. If anything, the critics of this question are mistaken in thinking that that recency of ancestry must be the sole basis for Linnaean classification.

Perhaps we can find a closer analogue of Simpson's criticism of logical typological thinking in the writings of Graham Budd and colleagues. Starting in the late 1990s, Budd has argued that an implicit tendency to associate phyla with rank‐defining, phylum‐level characters, has prompted “a logical flaw in attempts to talk about ‘phylum level evolution’” (Budd, 1999, p. 328). Budd sees this logical flaw manifested in presentations of evolution as a top‐down process:

If the taxonomic hierarchy truly reflected a “top‐down” pattern of evolution, so that “body plans” were suddenly generated, one would be entitled to ask what sorts of animals were being thus generated. If a proto‐mollusk suddenly emerged, one might want to inquire as to what the details of its morphology were – where it had sensory papillae or a shell, what shape its gills were, and so on. In this scenario, there could be no answer to these questions, because these would be ‘order’ or ‘family’ features, yet to emerge. (Budd, 1999, p. 328; also see Budd, 2013 and Budd & Jensen, 2000)

An error of this sort indeed *appears* to crop up in writings from the 1980s and 90s that Budd is concerned with, such as Valentine's big picture sketch of the Cambrian explosion: “Nearly all phyla appear in the Early Cambrian, body plans already in place so far as can be told, and then radiate into numbers of classes, and these into orders, so that the diversity peak of each lower taxonomic rank is shifted towards the present” (Valentine, 1994, p. 6751; also see Erwin, Valentine, & Sepkoski Jr, 1987 and Valentine, 1994). However, it would again be too rash to conclude from assertions like these that their authors are have committed a logical error. There are good reasons for thinking that the appearance of error is due to brevity of expression. Valentine could be read as saying that currently diagnostic features of many phyla (e.g., the radial symmetry and water vascular system of the *Echinodermata*; the segmented body, exoskeleton, and joint appendages of the *Arthropoda*) were already present by the early Cambrian and that features currently diagnostic of taxa of lower rank appear later in the fossil record. This hypothesis about the “top‐down” hierarchical nature of the origin of currently *diagnostic* features is perfectly compatible with holding that organisms that predated the evolution of those diagnostic features did belong to taxa at all main ranks. The reason why little has been said about the features of lower taxa is purely is epistemic: the resolution of the fossil record is too low to identify diagnostic criteria for extinct taxa at those ranks.

More recent work by Valentine suggests that something like this is what he meant all along. In *On the Origin of Phyla* (2004) he observes that, on the one hand, “the [fossil] record makes it appear that phyla evolved first; then classes evolved, … then orders evolved, … and so on,” but, on the other hand, he notes that “what might be expected during the origin of phyla … would be the divergences of two lineages from common ancestors, at first at the species level only. Then as time passed their differences would become more pronounced, the two lineages becoming as distinctive as are average genera, and then as are average families, then as orders, and so forth” (Valentine, 2004, p. 444). Valentine does not question the latter view. Therefore, the *appearance* of a top‐down formation of ranks must be just that: apparent, not real. It is an artifact of an incomplete fossil record, which hides from our view a buildup of morphological differences that preceded the assembly of body plans as we know them.

Finally, let us turn to the third, theoretical‐empirical error of typological thinking, according to which new taxa originate by saltation. Once again, the writings of Budd and colleagues contain attributions of this error, in close connection with the attributions of the logical error we just evaluated. Much like Simpson, Budd argues that an “over‐reliance on the taxonomic hierarchy as a guide to evolution” has been responsible for the development of “neo‐Goldschmidtian” models of the origination of taxa at the species level and above (Budd and Jensen, 1999, p. 327; also see Budd, 2006). For examples of these mistaken inferences from taxonomy to evolution Budd points to Arthur (2000), who speculated that systemic mutations and hopeful monsters could have played a role in the origin of body plans, and to Gellon and McGinnis (1998), who suggested that the origin of phyla may have been due to the sudden evolution of distinctive *Hox* gene suites.

Regardless of the merits of these saltationist hypotheses, it is not evident that they were triggered by mistaken ideas about the taxonomic hierarchy. Budd provides no direct support for his claim that the authors he cites actually made an error of logical typological thinking. He does, however, argue more generally that among paleontologists and evolutionary‐developmental biologists the “perception of systematics exerts a profound influence on evolution” in ways that hamper theorizing about the evolution of phyla (Budd, 2001, p. 487). The biased perception he is referring to consists in the failure to recognize the distinction between “crown groups” and “stem groups” of phyla.^2^

A phylum's crown group of is the group comprising all descendants of the common ancestor of the living members the phylum. Its stem group is composed of all extinct species that close the gap between the origin of the phylum and the origin of the crown group. Budd and Jensen (2000) argued that the failure to distinguish between crown groups and stem groups has constituted a “typological hindrance” toward our understanding of the origins of phyla. An impoverished conceptual repertoire has led many researchers to believe that “the essential features of body plans are forced suddenly to accumulate at the base of the new group, giving an emphasis to saltational notions of evolution” (Budd, 1999). Instead, the crown/stem group distinction allows that body plan features of phyla could have been assembled gradually along the stem groups. On this view, the phylum originates at the base of the crown group and its body plan becomes defined as the set of features that is plesiomorphically shared by all members of the crown group.

Although it is questionable whether failing to theorize in terms of stem and crown groups has indeed caused researchers to adopt saltationist views, it is evident that the renewed emphasis on the crown/stem group distinction has proven helpful in the subsequent discussions of the origins of phyla (Brysse, 2008).^3^ Even so, not everyone has agreed with how Budd and colleagues deploy this distinction to define phyla as crown groups. Briggs and Fortey (2005) and Collins and Valentine (2001) in particular have argued out that although Budd's crown group definition of phyla helpfully renders their delimitation a (cladistically) objective matter, this definition is ill‐suited for theorizing about the evolution of phyla. First, because it has the odd consequence of excluding the possibility of extinct taxa by definitional fiat. This is obviously undesirable from a paleontological perspective. Second, because restricting the definition of a phylum to the crown‐group entails that some species do not belong to a phylum at all, which gets us back to the same issues as we encountered in the discussion of Fitch and Sudhaus (2002). Finally, perhaps the most worrisome feature of the crown‐group definition of phyla is that the circumscription of a phylum and the identification of its body plan can become critically affected by chance survival of a marginal primitive species. If a species that is distantly related to other extant species in a crown‐based phylum goes extinct, this will radically change the time of origin of the phylum and may lead to the exclusion of many fossil species that now fall in the stem group. It seems odd to allow for a relative minor historical event to have such major implications: “There seems something very arbitrary about defining a major group on a whim of history” (Briggs & Fortey, 2005, p. 99).

The debate about how the crown/stem group distinction should be related to the definition and delimitation of phyla continues, with different conceptions of phyla being preferred by different researchers (e.g., see Budd, 2013; Erwin & Valentine, 2013). However, with the crown‐stem group distinction as such being on everyone's radar, the debate has advanced beyond the point where one could legitimately claim that certain researchers commit a fallacy of logical typological thinking. Continued assertions to this effect stand in the way of advancing genuine debates about the empirical and methodological constraints on what the study of development can tell us about macroevolution.

## 5 CONCLUSIONS

I have argued that the exposure and rejection of the Essentialism Story should not lead us to reject all attributions of typological thinking as meaningless or misguided. We can discern at least three meaningful “grades” of typological thinking that were implicit in Simpson's discussions of systematics before the image of typological thinking hardened into that of a metaphysical, Platonist doctrine under Mayr's influence. I have argued that attributions of these three grades of typological thinking—conceptual‐methodological, logical, and theoretical‐empirical—continue to feature in current debates about the nature and origins of phyla and phylum‐level body plans. However, while these attributions of typological thinking are meaningful as such, it is not always clear that they represent their contemporary targets accurately. The case Simpson made for several grades of typological thinking in his day does not transfer to today.

## ENDNOTES

1 This focus on recovering a forgotten *original* dimension of the typology/population distinction differentiates my approach from other *reformist* analyses of the typology/population distinctions, for example in application to the difference between character and character‐state evolution (Brigandt, 2007) or as involving different strategies of idealization and periodization (DiTeresi, 2010; Love, 2009).

2 See Dubois (2005) and Witteveen (2016b) for details on this nomenclatural “type method.”

3 The distinction was first proposed by Hennig (1969) and was developed (and given its current name) by Jefferies (1979).
